# Revisiting Research Methods in Language Learning Psychology From a Complexity Dynamic System Theory Perspective

**DOI:** 10.3389/fpsyg.2021.741045

**Published:** 2021-08-19

**Authors:** Jianbo Yang

**Affiliations:** Center for Second Language Writing Research, School of College English Teaching and Research, Henan University, Kaifeng, China

**Keywords:** language learning psychology, complexity dynamic system theory, data collection methods, research methods, complexity and dynamism

## Abstract

Language learning is a dynamic and complex process in which different factors and variables are constantly interacting. However, many studies done on constructs related to teacher-learner psychology have used one-shot quantitative research designs, while it is impossible to capture the complexity and dynamism of such variables *via* one-time measurements. Against this gap, complexity dynamic system theory (CDST) has recently been applied to explore processes and changes that a construct may undergo. To shed more light, the present study examined the current research methods used in this research domain and presented the contributions and different conceptualizations that can be made through CDST. In the end, some implications and future directions are suggested for passionate scholars.

## Introduction

Language learning is known as a complex process that requires different cognitive and linguistic skills and competencies to successfully occur (Larsen-Freeman and Cameron, 2008). When it is combined with psychology, emotions, and communication skills, this complexity is multiplied in human beings. Many of the underlying factors of language learning and learner's affect are in close relationships with other variables which are constantly interacting with each other (Xie and Derakhshan, 2021). The story gets more complicated when most of the factors and constructs involved in the language learning process develop through time and vary across contexts (MacIntyre et al., 2020). As a result, researching language learning, in general, and constructs related to learner-teacher psychology, in particular, are a demanding task that is impossible *via* traditional and simplistic approaches. To capture the dynamism of many variables in second language acquisition (SLA) and language learning psychology (LLP),scholars are recommended to use Complexity Dynamic System Theory (CDST) which grew out of Larsen-Freeman's (1997) seminal work.

The propositions of CDST have been widely applied in different fields such as humanities, social sciences, physics, natural sciences, psychology, and communication (Fogel, 2006; Wang et al., 2021). In contrast, SLA and applied linguistics have mainly used CDST as a theoretical lens instead of an empirical research basis (MacIntyre et al., 2017). As pinpointed by Larsen-Freeman (2017), the reason behind this gap is that in these two fields, running empirical studies on CDST has long been perceived as meta-theoretical in essence. That is to say, CDST is not a language learning theory, but instead an approach to develop a theory. Nevertheless, with the groundbreaking works of a number of SLA researchers (e.g., De Bot et al., 2007; Dörnyei et al., 2014; Larsen-Freeman, 2017; Hiver et al., 2021, among others), the applicability of this meta-theory in the field of SLA has been scientifically substantiated. Language education, owing to its inherent dynamism, variation, and stability, is a fertile ground to spread the seeds of CDST on. It goes well with CDST in that language learning and development is not solely a matter of language competence occurring in a vacuum, yet the offshoot of the interaction of numerous intervening factors which are continuously influencing each other as well as the language learning process.

Drawing on these conceptualizations, recently, SLA scholars have examined CDST in relation to the different learner and teacher psychology constructs including anxiety, motivation, willingness to communicate (WTC), agency, self-efficacy, demotivation, and enjoyment (Almutlaq and Etherington, 2018; Boudreau et al., 2018; Hiver and Papi, 2019; Larsen-Freeman, 2019; Syed and Kuzborska, 2020). In a similar manner, the impact of CDST on L2 students' listening (Dong, 2016), speaking (Dou et al., 2021), and writing skills (Fogal and Verspoor, 2020) has caught duly attention among researchers in this domain. However, most of the conducted studies in this area have collected their data without referring to the perspectives of CDST which can generate novel interpretations and methodological suggestions. Against this lack, the current study aimed to explain the research benefits of applying CDST to learner/teacher psychology and summons researchers for a revision in choosing their data collection methods.

## Background

### Complexity Dynamic System Theory (CDST)

CDST is a comprehensive approach to study the complicated systems and sub-systems of a phenomenon. It aims to unpack the underlying processes and factors which dynamically interact to cause an event, change, or development. This meta-theory has its roots in natural sciences such as biology, physics, chemistry, and mathematics where it has been implemented to justify simple systems (Amerstorfer, 2020). CDST takes a holistic approach to deal with dynamic and complex systems in which the incidents are no longer viewed as predictable, fixed, isolated, and in a simple cause-and-effect relationship (Larsen-Freeman, 1997). The theory brought fresh insights to SLA as its features were significantly fit with the tenets of CDST. The language was approved as a system in which numerous factors constantly interact and the collective outcome is the result of individual, intertwined components (Mercer, 2011). Based on this theory, language learning and development is no longer a linear, predictable, and identical process among all people. Instead, it is a complex system with many interconnected sub-systems which mutually affect each other in non-predictable trajectories (De Bot et al., 2007). In other words, language learning is the by-product of a network of personal, cultural, social, psychological, and contextual factors which are involved in a nested and co-adaptive relationship with blurry boundaries (Ushioda, 2015; Mercer, 2016). A tiny change in one of these components and sub-systems can drastically affect the whole system and cause unpredictable outcomes.

### Characteristics of CDST

As put by Mercer (2016), for a system to be considered as complex and dynamic some criteria must be met. These attributes that form the basis of CDST include; timescales, openness, predictability, stability, variability, attractor and repeller states, emergence, self-organization, soft assembly, fractalization, non-linearity, and sensitive dependence on initial conditions (the butterfly effect). ***Timescales***, as the most pivotal contribution, points to the effects of time on a process or attribute. Some constructs like anxiety, stress, and motivation may manifest themselves short-run, long-run, or even moment-by-moment. These timescales are nested in each other as seconds in minutes, minutes in hours, hours in days, days in weeks, weeks in months, months in years (Larsen-Freeman and Cameron, 2008). ***Openness*** refers to the impact of unpredicted sources on the functionality of a process or construct which brings clarity to their dynamics and unfolds their nature. ***Unpredictability*** of CDST argues that even by knowing the whole system and its interactions, one cannot predict the upcoming event or process. ***Stability*** concerns the relatively constant state of the overall system, for a period of time, despite the existing fluctuations. ***Variability*** of a system means that the current state of the system is the offshoot of the modifications to a previous state. ***Attractor*** state is what a system is doing at a particular moment whether it is pleasant or not. ***Repeller*** state is a state in which the system pushes something away regardless of its valence (positive or negative). ***Emergence*** proposes that the overall state of a system is something greater than the sum of its interacting components. ***Self-organization*** posits that systems do not follow a pre-specified plan but are intrinsically predisposed to organize themselves and show coherent patterns. ***Soft assembly*** suggests that the underlying components of a system can be re-configured into clear patterns as systems self-organize. Another feature of CDST is ***fractalization*** which refers to the ability of a system to exhibit and predict self-similar patterns/behaviors across various levels and timescales. ***Non-linearity*** suggests that the relationship between two factors in language learning is not always linear and straightforward. Finally, sensitive dependence on initial conditions or ***the butterfly effect*** argues that small features of the initial system can influence its subsequent evolution and functionality. Hence, small variations between two L2 students may entirely affect their language learning outcomes.

### The Nature of Research in CDST

Research in CDST perspective differs from simple quantitative and qualitative studies common in exploring teacher-learner psychology. The unique research properties of this meta-theory include; its substantially different *research questions* which are emergent and dynamically re-phraseable, *process-oriented approach* to understand an event considering one's cognition, emotion, social context, culture, and interactions, focus on *individual-experiences* rather than generalizable patterns of behavior, different *analytical tools*, and favoring *dense, longitudinal*, and *individual data* instead of large samples. Moreover, CDST research highlights ongoing and retrospective data collection techniques in place of self-report data in interviews, journals, and narratives to capture the dynamics of behavior. Likewise, CDST takes a holistic perspective which is against the reduction and fragmentation of factors and processes. It should be noted that referring to CDST as a conceptual framework to explain the data is not enough and it must be followed in the research design, data collection procedure, and data analysis phases.

## Researching LLP

Over the last decades, numerous studies have been conducted on different variables related to teacher-learner psychology mainly using one-shot and quantitative research designs (MacIntyre et al., 2020). Among many constructs in L2 learning, anxiety, motivation, stress, efficacy, WTC, interpersonal communication skills, resilience, stroke, enjoyment, engagement etc. have been substantially explored in different educational contexts (Dewaele and MacIntyre, 2014; Almutlaq and Etherington, 2018; Boudreau et al., 2018; Derakhshan et al., 2019; Derakhshan, 2021; Pishghadam et al., 2021). However, these studies have mostly been correlational and based on quantitative data. In scrutinizing LLP, some qualitative methods such as interviews, observations, and journals have also been employed to examine the process of change in these variables, but normally they have not captured the dynamism of change in action which is the heart of CDST.

It is evident that many of these constructs related to emotions and interpersonal communication skills (e.g., clarity, credibility, immediacy, rapport, and care) are unlikely to be measured *via* simple quantitative, one-shot, and pre/post-test designs. The nature of these variables in SLA is extremely complicated which takes a long period of time to be (re)constructed in a learner. Hence, the use of traditional quantitative-qualitative research designs falls short of scientific standards in this scholarly domain. That is why, when the same variables are examined by CDST, contradictory results may be obtained when the researcher scrutinizes the nature of a variable and its interacting sub-systems.

## The Applications of CDST to LLP Research

Despite its existing logistic challenges and the generalizability of findings, CDST has different applications to research in SLA and LLP. As a case in point, researchers can investigate constructs related to teacher-learner psychology focusing on different timescales to see if the observed patterns of behavior occur at other levels. Similarly, CDST provides fresh insights for running idiodynamic studies to explore the nature of dynamic, stable, and variable changes in a construct (Boudreau et al., 2018). Moreover, this theory urges avid scholars to focus on unusual, unpredictable, unique, and outlier cases. They can also integrate social and cognitive dimensions of SLA using CDST (Larsen-Freeman, 2010). Additionally, CDST opposes a priori determination and definition of constructs and their measurements and stresses that LLP constructs must be put in motion to see how they interact and go together leaving a space for the potential influences from other sources on their development. Methodologically, CDST has introduced novel quantitative and qualitative research methods to applied linguistics including panel designs, latent growth curve modeling, multilevel modeling, idiodynamic method, qualitative comparative analysis, process tracing, retrodictive qualitative modeling, agent-based modeling, and socialnetwork analysis (Hiver and Al-Hoorie, 2020). Each of these methods can significantly capture the complexity and dynamism of LLP variables. Nevertheless, CDST is still a nascent approach to research the dynamics of teacher-learner psychology which requires complementary studies at various levels to have substantial contributions. Otherwise, it fades away as time passes, and if nothing valuable is offered by the theory.

## Implications and Future Directions

In this study, it was concluded that language learning psychology is a complicated phenomenon that is not measurable *via* one-shot research instruments. To capture its complexity and dynamism, CDST approach was introduced to SLA and LLP research to detect the developmental process of many social, cognitive, and psychological teacher-learner variables. Although this theory seems technical, complicated, and with logistic problems, its application to L2 education is advantageous in that it can increase the awareness of L2 teachers, students, materials developers, and researchers. Teachers can use the findings to improve students' language learning by using teaching methods and classroom activities that are in tune with the principles of CDST. Similarly, students would find this study of value in that they can realize that language learning is the result of an interplay of numerous interacting components and takes a logical time to develop. Materials developers can develop textbooks and activities which reflect the dynamism and non-linearity of language competence. Finally, researchers can use the ideas of this study to run comparable studies in other fields and contexts as CDST is context-specific. Moreover, running cross-cultural studies is a fresh topic for research to see the applicability of CDST in different cultures. Additionally, future studies can be conducted on teacher-related and interpersonal communication variables from the perspective of CDST. Likewise, emotions in language education can be explored longitudinally through the lens of CDST. Finally, CDST research designs can be employed by future scholars to drive the field forward regarding the dynamics of SLA.

## Author Contributions

JY independently drafted the first manuscript and revised meticulously before it was submitted to this special issue for Frontiers in Psychology.

## Conflict of Interest

The author declares that the research was conducted in the absence of any commercial or financial relationships that could be construed as a potential conflict of interest.

## Publisher's Note

All claims expressed in this article are solely those of the authors and do not necessarily represent those of their affiliated organizations, or those of the publisher, the editors and the reviewers. Any product that may be evaluated in this article, or claim that may be made by its manufacturer, is not guaranteed or endorsed by the publisher.
